# Cyclodextrin Nanosponge–PVA Hybrid Networks as Supramolecular Reservoir Systems for Amitriptyline

**DOI:** 10.1021/acsomega.6c03745

**Published:** 2026-07-16

**Authors:** Bianca B. M. Vieira, Pedro M. C. Matias, Dina M. B. Murtinho, Eduardo Radovanovic, Frederico B. De Sousa, Artur J. M. Valente

**Affiliations:** † Laboratório de Sistemas Poliméricos e Supramoleculares (LSPS) − Instituto de Física e Química, Universidade Federal de Itajubá (UNIFEI), Itajubá 37500-903, MG, Brazil; ‡ CQC-IMS, Department of Chemistry, Coimbra University, Coimbra 3004-535, Portugal; § Departamento de Química, Universidade Estadual de Maringá, Maringá 87020-900, PR, Brazil

## Abstract

Poly­(vinyl alcohol) (PVA) hydrogels incorporating β-cyclodextrin (βCD) nanosponges (CDNSs) were developed as hybrid supramolecular systems for the controlled delivery of amitriptyline hydrochloride (AMT). The βCD-derived nanosponges provide host–guest cavities for drug inclusion, while the PVA network acts as a diffusion-regulating matrix. Initially, adsorption–desorption studies were performed on isolated CDNSs to evaluate their drug-loading capacity prior to incorporation into the hydrogels. The CDNSs exhibited efficient encapsulation (∼60%) and released more than 90% of the drug, confirming strong yet reversible host–guest interactions within CDs cavities. The drug-loaded CDNSs were subsequently dispersed in PVA solutions and physically cross-linked by freeze–thaw cycling, producing hydrogels containing embedded supramolecular reservoirs. Increasing the PVA concentration (from 14 to 20% w/v) generated denser polymer networks, reducing the swelling degree from ∼60 to ∼30 and slowing water uptake. CDNSs incorporation altered the hydrogel microstructure and introduced additional drug–matrix interactions, resulting in reduced burst release, measurable lag times, and more prolonged release compared with neat PVA hydrogels. Release kinetics were best described by the Weibull model, indicating diffusion-controlled transport in the pure matrices and heterogeneous diffusion behavior in the hybrid systems. Overall, AMT release is governed by coupled mechanisms involving diffusion through the polymer network and dissociation from cyclodextrin host sites, with CDNSs acting as supramolecular reservoirs within the hydrogel matrix.

## Graphical abstract

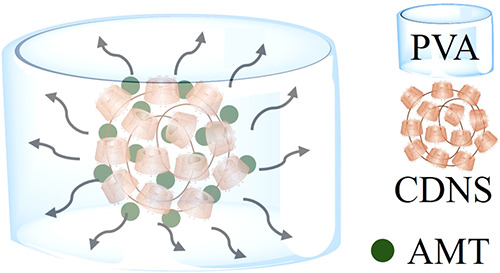

## 1 Introduction

The 3-(10,11-dihydro-5H-dibenzo­[*a*,*d*]­cycloheptene-5-ylidene)-*N*,*N*-dimethylpropan-1-amine, also known as amitriptyline (AMT), is a tricyclic drug (Figure 1a) that exhibits significant efficacy in the treatment of depression, contributing to improvements in sleep and fatigue, and, as an analgesic, in several pathologies associated with significant pain, such as fibromyalgia.^1,2^ Among other applications, it has recently been demonstrated that AMT also plays an effective role in the prevention and treatment of migraine.^3^ AMT is characterized by a p*K*_a_ equal to 9.4, showing that the hydrochloride will primarily exist in the protonated form in the majority of cases.^4^ Notwithstanding, the first-pass metabolism may require overdosing to ensure adequate bioavailability, which may lead to side effects, such as anticholinergic activity and interference with potassium channels.^5,6^

**Figure 1:**
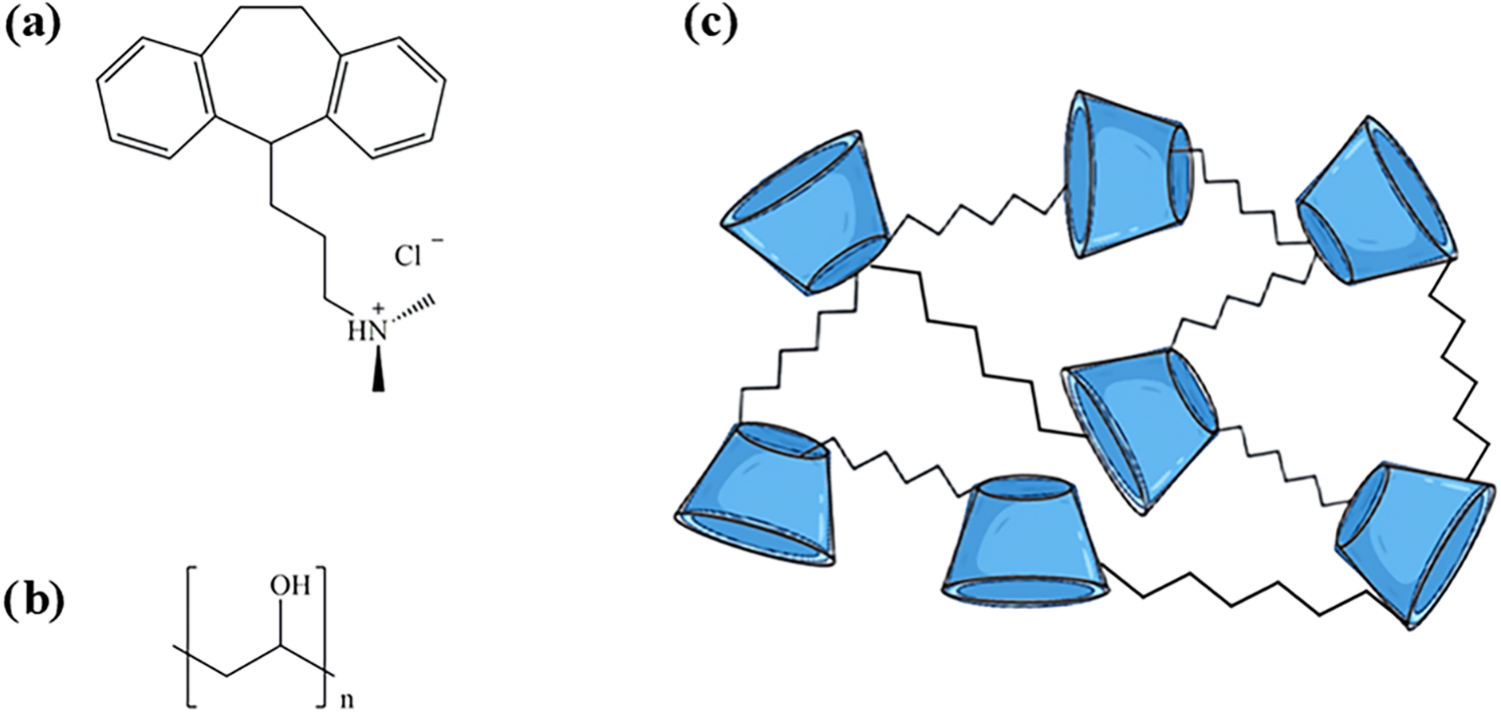
(a) Structural formula of amitriptyline hydrochloride; (b) structural formula of the PVA monomer; and (c) schematic representation of a CDNS.

Different types of matrices and delivery systems have been developed to overcome some of the drawbacks faced by drugs such as AMT, including electrospun polymeric fibers,^7^ nanoparticles,^8^ liposomes,^9^ micelles,^10^ and hydrogels.^11^ The latter are widely used due to their swelling capacity, versatility in incorporating different bioactive molecules, and ease of production. Additionally, hydrogels can provide an aqueous microenvironment similar to that of biological tissues, ensuring biocompatibility.^12^ Among the various polymers, poly­(vinyl alcohol) (PVA) (Figure 1b) stands out due to its notable properties, including biocompatibility, the ability to form stable freeze–thaw networks, and an amphiphilic nature.^13,14^ Despite these advantages, hydrogels often face limitations in applications involving the release of active substances, such as rapid diffusion of loaded drugs or uncontrolled degradation of the polymeric matrix. To overcome these drawbacks, kinetic modulation strategies have been developed, including adjustments in cross-linking density^15^ or the incorporation of dual-encapsulation systems.^16^ In dual-encapsulation systems, the drug is first encapsulated into nanocarriers that are subsequently embedded within the hydrogel. This approach enables more precise control over release kinetics, reduces the burst-release effect, and maintains more stable therapeutic levels.^17,18^

Among the available nanocarriers, cyclodextrin-based systems stand out owing to their structural versatility and ability to form supramolecular host–guest complexes (HGcs).^19^ Cyclodextrins (CDs) are cyclic oligosaccharides characterized by a less hydrophilic inner cavity and a hydrophilic outer surface, which confer amphiphilic properties.^20,21^ The obtained HGcs enhance the solubility, stability, and bioavailability of guests from different classes while also protecting them against chemical degradation and undesirable interactions.^22,23^ More recently, the chemical cross-linking of CDs has led to the development of cyclodextrin-based nanosponges (CDNSs) (Figure 1c), an innovative class of highly cross-linked and nanoporous polymers. In the literature, a wide variety of strategies for obtaining these cross-linked and nanoporous polymers have been reported,^24^ including the use of diamines with different alkyl chain lengths, which allows us to significantly alter both in terms of CD mobility and provides methylene groups that may induce additional interactions with the encapsulated species.^25^ These three-dimensional structures combine the biocompatibility and low cytotoxicity of CDs with enhanced structural robustness, offering multiple sites for drug interaction, both within the CD cavities and across the polymeric network.^26–30^

The incorporation of CDNSs into PVA hydrogels integrates the advantages of both systems: while the nanosponges act as reservoirs that protect and gradually release the drug,^28,31^ the hydrogel provides structural support, biocompatibility, and water retention, thereby creating a favorable microenvironment for controlled diffusion.^32,33^ This hybrid strategy not only minimizes the burst-release effect, a typical limitation of conventional hydrogels, but also optimizes drug encapsulation capacity and expands the potential for application across different administration routes.^33–35^

In this work, we aim to prepare hybrid blend materials based on PVA hydrogels containing CDNSs for the loading and release of an antidepressant, AMT. This strategy combines the advantages of both polymeric systems by enabling an improved reservoir for drug protection and release,^36,37^ while providing structural support, biocompatibility, and water retention, thereby creating a favorable microenvironment for controlled diffusion.^32,37^ To this end, two βCD-based CDNSs functionalized with amines were synthesized and characterized. The encapsulation and release efficiencies, as well as the loading capacity of AMT.HCl in these materials, were investigated as a preliminary step to assess their drug-loading and release potential. Subsequently, the drug-loaded CDNSs were incorporated into PVA hydrogels and evaluated.

## 2 Experimental Section

### 2.1 Reagents and Solvents

For the synthesis of CDNSs, βCD (>98.0%, CAS 7585–39–9), triphenylphosphine (PPh_3_, ≥99%, CAS 603–35–0), and iodine (≥99.8%) were purchased from Sigma-Aldrich, Thermo Scientific, PanReac Applichem, respectively. Dodecane-1,12-diamine (am_12_, ≥98%, CAS 2783–17–7) and hexane-1,6-diamine (am_6_, 98%, CAS 124–09–4), used as cross-linkers, were purchased from Tokyo Chemical Industry (TCI) and Sigma-Aldrich, respectively. *N*,*N*-Dimethylformamide (DMF, ≥99.8%, CAS 68–12–2) and dimethyl sulfoxide (DMSO, ≥99.9%, CAS 67–68–5) were supplied by Honeywell and Sigma-Aldrich, respectively, and were dried using molecular sieves. Methanol (MeOH, HPLC grade, CAS 67–56–1) and diethyl ether (≥99.8%, CAS 60–29–7) were provided by Chem Lab and Honeywell, respectively. For ^1^H NMR experiments, deuterated dimethyl sulfoxide (DMSO-*d*_6_, purity 99.8%, CAS 67–68–5) was purchased from Eurisotop and used as the solvent. For adsorption assays, AMT (≥98%, CAS 549–18–8) was used, purchased from Sigma-Aldrich. For the preparation of the hydrogels, PVA (*M*_W_ ∼ 61,000, degree of hydrolysis 98.0–98.8 mol %, CAS 9002–89–5) was obtained from Sigma-Aldrich. Organic solvents were purified or dried prior to use, following standard procedures. Other commercially available solvents were used without further purification.

### 2.2 Synthesis of Amine-Cyclodextrin Nanosponges

The CDNSs were obtained through a reaction between βCDs functionalized with amine groups (am_*x*_-βCD) and iodinated βCD (I-βCD) (Figure 2). The I-βCD was previously prepared by modifying βCD via a nucleophilic substitution reaction using an excess of I_2_, as previously described.^38^ In brief, Ph_3_P (30.0 mmol) is initially dissolved in 30 mL of anhydrous DMF. Subsequently, I_2_ (31.37 mmol) was gradually added to this solution until complete dissolution. βCD (2.0 mmol) was then added to the mixture. The resulting mixture was stirred under a nitrogen atmosphere at 70 °C for 21 h. After that, the reaction was cooled to r.t. and concentrated by reducing the solvent volume (∼50%) using a rotary evaporator. Then, a freshly prepared solution of NaOMe (3.0 mol·L^–1^, 12 mL) was added and stirred under cooling for 30 min. The reaction mixture was then poured into an excess of MeOH, and the precipitate was collected by vacuum filtration. The solid was briefly washed and extracted in a Soxhlet apparatus with MeOH until no color was detected in the solvent (98% yield).

**Figure 2:**
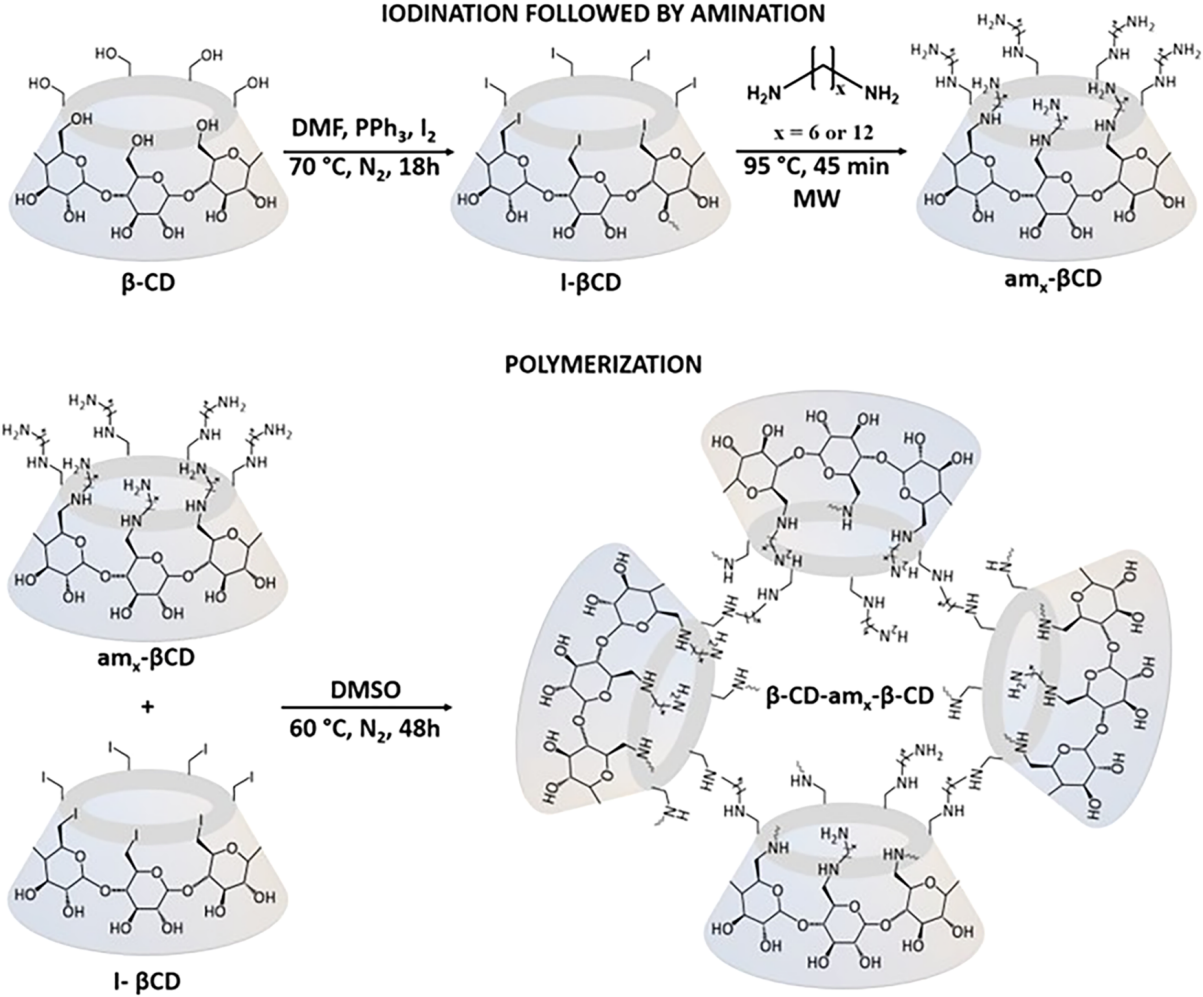
Scheme representing the reactions performed for the synthesis of the nanosponges: iodination, amination, and polymerization between I-CD and am_*x*_-CD derivatives.

Starting from I-βCD, the am_*x*_-βCDs (am_12_-βCD and am_6_-βCD) were prepared by nucleophilic substitution of the iodine atom at the C6 position of I-βCD by using two diamines, dodecane-1,12-diamine (am_12_) and hexane-1,6-diamine (am_6_). The syntheses were carried out using the previously described procedure;^39^ however, in this case, the reaction took place in a CEM DISCOVER microwave reactor, using a fixed temperature method at 95 °C, for 45 min under 10 bar pressure (am_12_-βCD 78% yield and am_6_-βCD 96% yield).

Finally, the synthesis of the cyclodextrin-based nanosponges functionalized with amine groups (βCDam_12_βCD and βCDam_6_βCD) was carried out by reacting I-βCD with βCDam_12_ and βCDam_6_, respectively, using a 1:1 molar ratio. The reagents were mechanically mixed, and anhydrous DMSO (250 mL) was used as the medium to facilitate reagent homogenization. The mixtures were heated at 60 °C for 48 h under stirring. After 48 h, the resulting brown gum-like product was transferred to a beaker containing 10 mL of distilled water. The solid was collected by centrifugation at 8000 rpm for 20 min. Three washing cycles were performed by sonicating the solid in 10 mL of methanol for 10 min each, followed by a final wash with 10 mL of diethyl ether. The product was collected by centrifugation and stored in a desiccator until further use (βCDam_12_βCD 159 mg and βCDam_6_βCD 331 mg).

### 2.3 Characterization of Materials

The synthesis of I-βCD and the amine-functionalized βCDs (am_12_-βCD and am_6_-βCD) was followed by nuclear magnetic resonance spectroscopy (^1^H NMR) through a Bruker AVANCE III HD 400 MHz spectrometer.

Fourier-transform infrared spectroscopy (FTIR) was performed in a Thermo Scientific Nicolet 6700 spectrometer (with KCl pellets), in the wavenumber range of 4000–400 cm^–1^ and in a Thermo Scientific Nicolet 380 spectrometer with an ATR accessory in the range of 4000–650 cm^–1^. All spectra were recorded with 64 scans per sample and a spectral resolution of 4 cm^–1^.

The thermal stability of the compounds was determined by thermogravimetric analysis (TGA/dTG) using a TG209 F3 thermogravimetric analyzer (Netzsch Tarsus). The analysis was performed using 3.0 mg of sample under a nitrogen atmosphere with a flow rate of 50 mL min^–1^. Samples were heated in an alumina crucible over a temperature range of 20–600 °C, employing a heating rate of 10 °C min^–1^. TG/DTG curves were obtained for βCD, I-βCD, am_6_βCD, am_12_βCD, as well as for βCD-am_6_-βCD and βCD-am_12_-βCD.

Dynamic light scattering (DLS) and ζ-potential measurements were performed using a Zetasizer ZS NanoSeries instrument (Malvern Instruments) with a folded capillary zeta cell (Malvern Panalytical, DTS1070). Measurements were carried out using 1.0 mg of each CDNS dispersed in 2.0 mL of Milli-Q water at room temperature, across a pH range of 2–12, and a detection angle of 173°. Samples were subjected to a He–Ne laser beam with a wavelength of 632 nm, and the data were analyzed using Zetasizer software version 8.02. The pH was adjusted by microcontrolled addition of aqueous solutions of HCl or NaOH (0.01 mol L^–1^).

The specific surface area of the CDNSs was determined by nitrogen adsorption–desorption (Micromeritics ASAP 2000), applying the Brunauer–Emmett–Teller (BET) model to calculate the pore size.

The surface morphology of the CDNSs was evaluated by using scanning electron microscopy (SEM) (JEOL model JSM 5310). Micrographs were obtained at 15 kV, both before and after contact with 40.0 mmol L^–1^ aqueous AMT solutions. For hydrogels, they were previously frozen in liquid nitrogen and lyophilized for 24 h (FreeZone 4.5, Labconco). All samples were coated with a gold layer.

### 2.4 Adsorption Experiments

Adsorption experiments were carried out by placing 1.0, 10.0, and 50.0 mg of each material in contact with 1.0 mL of an aqueous solution of AMT (40.0 mmol·L^–1^). The samples were kept in an ultrasonic bath for 8 h at approximately 45 °C. The amount of AMT adsorbed was quantified by UV–vis absorption spectroscopy (Shimadzu UV-2600i) at 239 nm, using the following molar absorption coefficient: ε = 13,839 (±144) L·mol^–1^·cm^–1^. After quantifying the encapsulated drug, the encapsulation efficiency (EE) and loading capacity (LC) of materials were calculated using eqs ([Disp-formula eq1]) and ([Disp-formula eq2]), respectively:

1 $$\mathrm{EE}\left(\%\right)=\left(\frac{W_{0}-W_{f}}{W_{0}}\right)\times 100$$

2 $$\mathrm{LC}\left(\%\right)=\left(\frac{W_{0}-W_{f}}{W_{m}}\right)\times 100$$

where *W*_0_ and *W*_f_ are the initial and final amounts of drug in the aqueous solution, respectively, and *W*_m_ is the total weight of the drug-loaded material.

Subsequently, the potential of the materials for drug release was evaluated through AMT desorption experiments. For this purpose, after the adsorption experiments, the polymers were immersed in 10 mL of Milli-Q water and kept under agitation in an incubator (150 rpm) for 70 h at 25 °C. Drug quantification was performed as previously described. The release efficiency (RE) was calculated using eq ([Disp-formula eq3])

3 $$\mathrm{RE}\left(\%\right)=\left(\frac{W_{t}}{W\mathrm{enc}}\right)\times 100$$

where *W*_t_ is the weight of the drug released into the aqueous medium and *W*_enc_ is the weight of the drug previously encapsulated in the polymers.

### 2.5 Preparation and Characterization of PVA Hydrogels

PVA hydrogels were prepared at concentrations of 14 and 20% w/v, both pure and loaded with the drug AMT. In addition, PVA hydrogels (20% w/v) were prepared containing the AMT:βCD complex (see Supporting Information (S1)) and CDNSs already loaded with AMT (labeled as βCD-am_6_-βCD:AMT and βCD-am_12_-βCD:AMT), using the freeze–thaw method.^40^ An accurate mass of the material was dissolved in 1.0 mL of the PVA solution and left to stir (120 rpm) for 1 h. After that, the solutions were poured into Petri dishes and subjected to freezing at −20 °C for 12 h, followed by thawing at r.t. for another 12 h. The freeze–thaw cycles were repeated three times.

### 2.6 Swelling Degree

The swelling degree of PVA-based hydrogels in water was obtained by weighing the samples before and after immersion in water, following procedures previously described in the literature.^40,41^ Each hydrogel was weighed and subsequently immersed in Milli-Q water. The swelling degree was calculated as the average of three independent measurements using eq ([Disp-formula eq4])

4 $$Q_{s}=\frac{\left(W_{s}-W_{x}\right)}{W_{x}}$$

where *Q*_s_ is the swelling degree, *W*_s_ is the weight of the swollen hydrogel, and *W*_x_ is the weight of the xerogel. The xerogel weight was determined based on the initial weight of the sample prior to swelling, considering the solid content of the gel. The data were analyzed by using a two-sample Student’s *t* test, assuming unequal variances and a significance level of 5% (α = 0.05). To assess the water sorption mechanism, swelling kinetics were investigated by applying different kinetic models (detailed in Supporting Information S2). The goodness of the fitting was assessed by using the coefficient of determination (*R* ^2^) and the Akaike information criterion (AIC), eq ([Disp-formula eq5])

5 $$\mathrm{AIC}=n\;\log\left(\frac{s^{2}}{n}\right)+2K$$

where *n* is the number of experimental data points, *s* ^2^ is the residual sum of squares, and *K* is the number of model parameters.

### 2.7 Kinetics Release Study

For the AMT release kinetics, AMT-containing hydrogel samples were immersed in 200 mL of water at 25 °C and 150 rpm. The released amount of AMT was measured by UV–vis spectrophotometry, at every 3 min, over 12 h, by using an automatic sampling Hsensor (Equipamentos, Materiais e Acessórios LTDA, Brazil). A full statistical analysis was performed to evaluate whether any difference between groups is significant.

## 3 Results and Discussion

### 3.1 Synthesis of Amine-Cyclodextrin Nanosponges

The structures of βCD, I-βCD, am_6_-βCD, and am_12_-βCD were confirmed by ^1^H NMR, and the following data were obtained: **βCD**: ^1^H NMR (400 MHz, (CD_3_)_2_SO) δ 5.72 (d, *J* = 6.9 Hz, 7H), 5.67 (d, *J* = 2.5 Hz, 7H), 4.84 (d, *J* = 3.6 Hz, 7H), 4.45 (t, *J* = 5.6 Hz, 7H), 3.72–3.53 (m, 28H), 3.36–3.24 (m, 14H); **I-βCD**: ^1^H NMR (400 MHz, (CD_3_)_2_SO) δ 6.02 (d, *J* = 6.9 Hz, 7H), 5.92 (d, *J* = 2.3 Hz, 7H), 4.99 (d, *J* = 3.9 Hz, 7H), 3.81 (dd, *J* = 10.9, 2.2 Hz, 7H), 3.71–3.52 (m, 14H), 3.52–3.35 (m, 14H), 3.28 (d, *J* = 9.8 Hz, 7H); **am**_**6**_ **-βCD**: ^1^H NMR (400 MHz, (CD_3_)_2_SO) δ 4.84 (s, 7H), 3.57–3.30 (m, 42H), 2.81–2.56 (m, 28H), 1.49–1.18 (m, 56H); **am**_**12**_ **-βCD**: ^1^H NMR (400 MHz, (CD_3_)_2_SO) δ 4.81 (s, 7H), 3.71–3.42 (m, 35H), 3.30 (d, *J* = 9.0 Hz, 7H), 2.82–2.54 (m, 28H), 1.49–1.13 (m, 140H). All assignments are consistent with the literature.^38,39^ Precursors and polymers were further characterized by FTIR spectroscopy. The spectra of the precursors (βCD, I-βCD, am_6_, and am_12_) are shown in Figure S1, while the corresponding assignments of the main vibrational modes are presented in Tables S1 and S2. The obtained spectra are in close agreement with those reported elsewhere.^42,43^ It should be noticed that the substitution of primary hydroxyls at the C6 position by iodine atoms can be followed by the vibrational mode occurring at 3348 cm^–1^. The presence of iodine in the structure is also confirmed by the appearance of well-defined bands in the regions of 1200 and 586 cm^–1^, which can be attributed to CH_2_–I bending and C–I, stretching, respectively.^44^

The FTIR spectra of am_6_-βCD, βCD-am_6_-βCD, am_12_-βCD, and βCD-am_12_-βCD are presented in Figure 3. In these spectra, the main vibrational mode characteristic of βCD is also observed. The amination process (am_*x*_-βCD) results in a broadening of the band around 3350 cm^–1^, which can be attributed to the contributions of the vibrational modes of the amine groups, indicating that the ν­(N–H) bands of primary and secondary amines are superimposed on the band assigned to the ν­(O–H) of CD.^45^ Additionally, the spectra of am_6_-βCD and am_12_-βCD display two well-defined bands around 2900 cm^–1^, assigned to the ν­(C–H) and (C–H_2_) stretching of the aliphatic chain. Amination is also confirmed by the presence of a well-defined band ca. 1650 cm^–1^, corresponding to δ­(N–H), observed in both the am_6_-βCD and am_12_-βCD spectra. When comparing the spectra of am_6_-βCD and am_12_-βCD with those of respective CDNSs (Figure 3a,3b), the presence of all bands related to am_*x*_-βCD can be observed; however, there is a significant decrease in band intensities, which may be associated with the cross-linking effect.^39^

**Figure 3:**
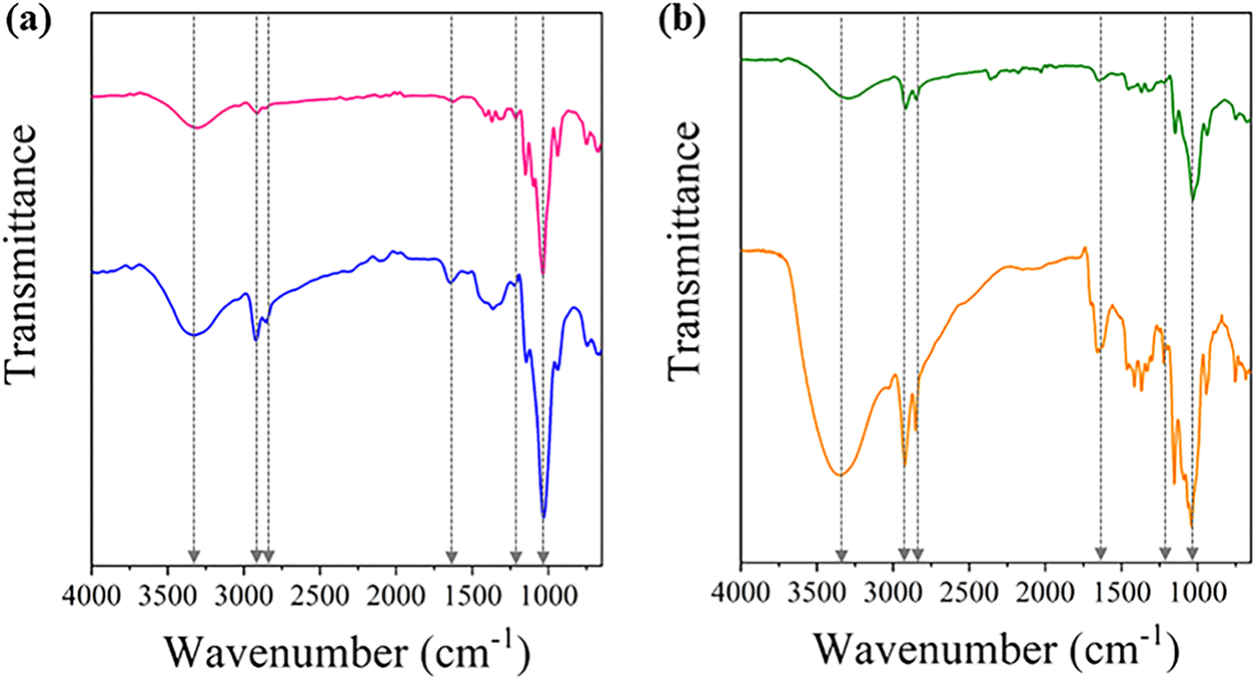
FTIR-ATR spectra of (a) βCD-am_6_-βCD (pink), am_6_-CD (blue) and (b) βCD-am_12_-βCD (green), am_12_-βCD (orange).

Thermal gravimetrical analysis of precursors and polymers was carried out to better understand the effect of polymerization on the thermal stability of polymers, and data are shown in Figures 4, S2 and Table 1. For all the compounds, an initial mass loss is observed in the range of 25 to 100 °C, corresponding to solvent evaporation. The thermal decomposition profile of βCD is consistent with that reported in the literature, showing a maximum degradation rate temperature (*T*_max_) around 313 °C, with a mass loss of 86% (250–370 °C).^46,47^ After the iodination process (I-βCD), *T*_max_ decreased to 239 °C. The decrease in *T*_max_ can be justified by the decrease in the number of intra- and intermolecular hydrogen bonds.^39,46^

**Figure 4:**
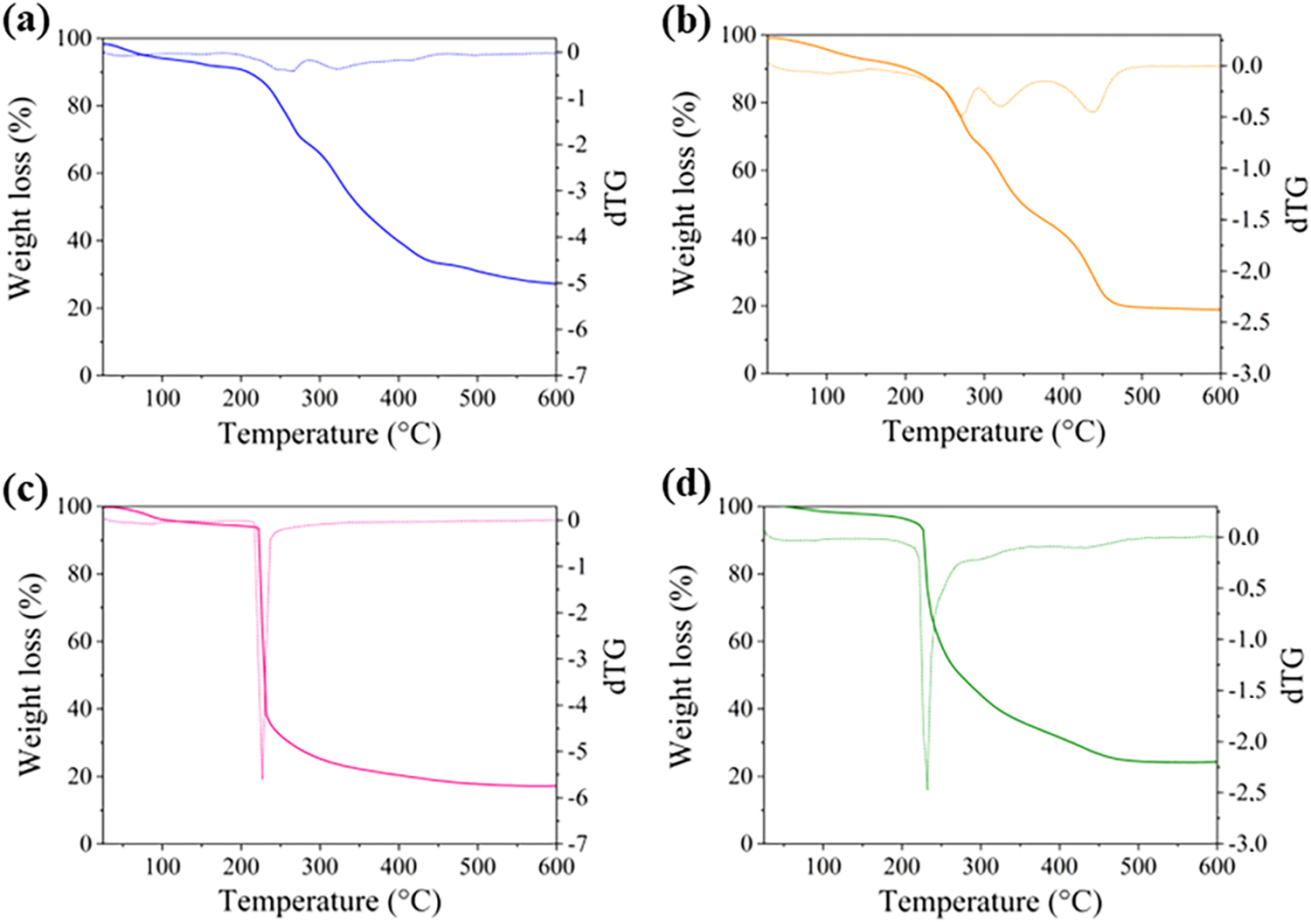
Thermograms (solid line) and corresponding derivatives (dTG, dash dot line) for (a) βCD-am_6_; (b) βCD-am_12_; (c) βCD-am_6_-βCD and (d) βCD-am_12_-βCD.

**Table 1:** TG and dTG Data of βCD, I-βCD, am_6_-βCD, am_12_-βCD, βCD-am_6_-βCD, and βCD-am_12_-βCD

	temperature range (°C)	*T*_max_ (°C)	weight loss (%)
βCD	(1) 250–370 °C	(1) 313 °C	(1) 86.0%
I-βCD	(1) 223–270 °C	(1) 239 °C	(1) 57.4%
am_6_-βCD	(1) 174–285	(1) 263	(1) 23.3%
	(2) 285–368	(2) 320	(2) 22.2%
	(3) 368–453	(3) 412	(3) 12.8%
am_12_-βCD	(1) 165–293	(1) 272	(1) 24.5%,
	(2) 293–375	(2) 322	(2) 22.3%
	(3) 375–488	(3) 438	(3) 25.9%
βCD-am_6_-βCD	(1) 206–285	(1) 227	(1) 68.4%
βCD-am_12_-βCD	(1) 206–285	(1) 232	(1) 46.8%
	(2) 285 –341	(2) 298	(2) 10.4%
	(3) 341–496	(3) 438	(3) 22.8%

Both aminated CDs, am_6_-βCD and am_12_-βCD, exhibit similar thermal behavior, with three degradation steps—excluding the solvent evaporation (Figure 3a,3b). Two thermal events, *T*_max(1)_ and *T*_max(2)_, correspond to the two-step decomposition of the CDs, while a third one, *T*_max(3)_, may be associated with the cleavage of the secondary amine bond at the C6 position.^25,39^ Comparing the *T*_max_ values, am_12_-βCD shows slightly higher values, which can be attributed to the longer carbon chain of the am_12_ diamine, which has a higher compaction ability due to van der Waals and hydrophobic interactions. Additionally, the initial decomposition temperatures of the aminated CDs are higher than those observed for I-βCD. This behavior suggests that the amination process confers greater thermal stability, possibly due to the increased hydrogen bonding provided by the incorporation of primary and/or secondary amine groups.^25,39^

The CDNSs exhibit distinct thermal decomposition profiles (Figure 4c,4d). βCD-am_6_-βCD shows a single decomposition step, suggesting a more homogeneous material structure,^25^ whereas βCD-am_12_-βCD displays multiple steps, indicating a more heterogeneous structure, in agreement with the literature.^25,48^ Furthermore, both CDNSs have lower *T*_max_ values than those observed for their respective aminated CDs and the native CD, indicating a reduction in thermal stability following the cross-linking process.

The data obtained by DLS for βCD-am_6_-βCD and βCD-am_12_-βCD are summarized in Tables S3 and S4, respectively, detailing the intensity- and number-based distributions, over the pH range of 2–12. Analysis of the data revealed that pH influences particle size. At certain pH values, bimodal populations were observed, while at others, only a single population was detected, with no clear trend across the studied pH range. In cases where two populations coexist (Figure 5a), the number-based distribution analysis showed that the smaller particles are predominant, representing more than 80% of the total in both CDNSs.

**Figure 5:**
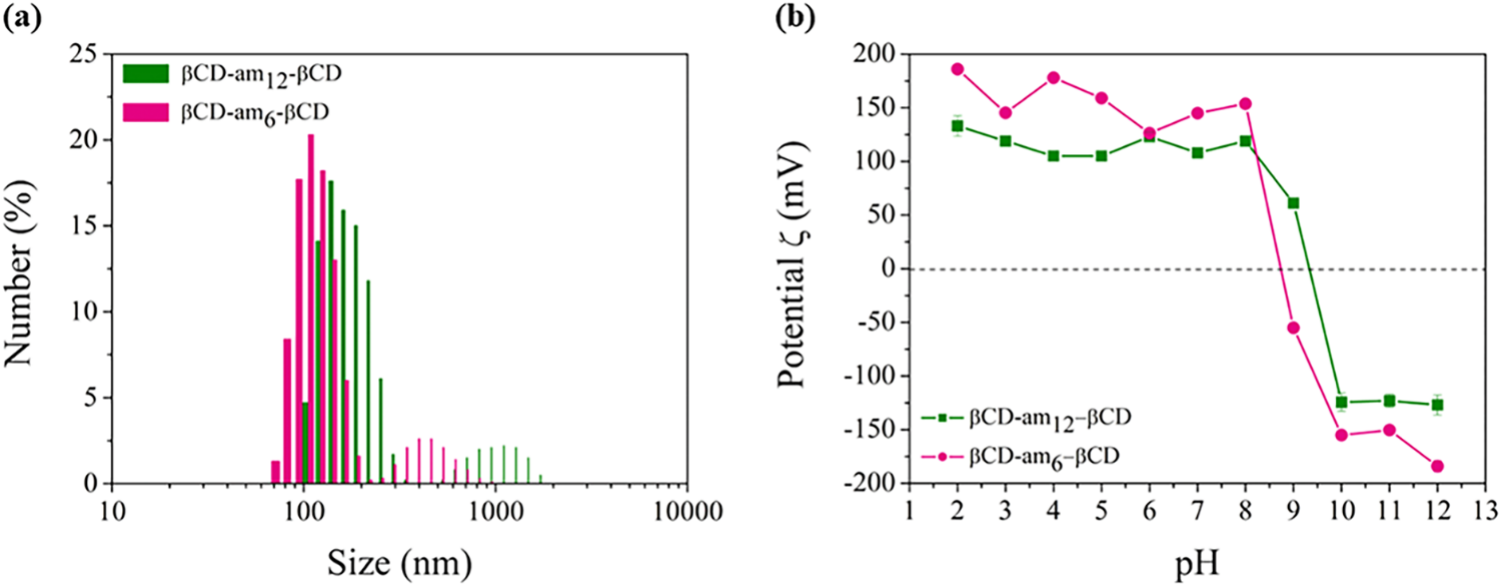
(a) Particle size distribution obtained from DLS of βCD-am-_12_βCD and βCD-am_6_-βCD, at pH 7, and (b) effect of pH on the ζ-potential for (■) βCD-am-_12_βCD and (●) βCD-am_6_-βCD.

Both CDNSs exhibited PDI values higher than 0.1 for all pH values, indicating the occurrence of polydisperse systems. In particular, PDI values for βCD-am_12_-βCD approached 1 at several pH values, suggesting a highly polydisperse system. This behavior is consistent with the thermogravimetric data, which indicate multiple decomposition steps for βCD-am_12_-βCD, pointing to a more heterogeneous material compared to βCD-am_6_-βCD.^49^

The surface charge of the particles was evaluated by ζ-potential analysis (Figure 5b). Both CDNSs exhibited a positive ζ-potential in acidic and slightly basic media, specifically between pH 2 and 8 for βCD-am_6_-βCD and between pH 2 and 9 for βCD-am_12_-βCD. This behavior suggests that the primary and secondary amine groups present in the structures are protonated within these pH ranges. The isoelectric points (IEPs) were determined based on the ζ-potential curves, observed at pH 8.7 for βCD-am_6_-βCD and pH 9.3 for βCD-am_12_-βCD. Above these values, both CDNSs display a negative charge due to amine group deprotonation. Following this, the surface morphology of βCD-am_12_-βCD presents a rough structure of greater homogeneity throughout the analyzed area, unlike the βCD-am_6_-βCD, which presents a significant heterogeneity in the morphological pattern, suggesting that there are aggregation zones indicated in the featureless surface zones (Figure 5). Interestingly, the N_2_ adsorption has shown that the BET surface area for βCD-am_6_-βCD is higher than that for βCD-am_12_-βCD (23.2 m^2^/g and 0.33 m^2^/g, respectively). These values are consistent with higher values for the latter (55.3 nm) compared with the former (19.9 nm).

### 3.2 Adsorption Experiments

Adsorption experiments were conducted using the drug AMT to investigate the potential interaction between the drug and the synthesized CDNSs (βCD-am_6_-βCD and βCD-am_12_-βCD). These studies aimed to evaluate the feasibility of encapsulating AMT within materials. The EE% and LC% of AMT in the CDNSs, using different amounts of nanosponges, are presented in Figure 7a.

From the analysis of Figure 7a, we can conclude that the encapsulation efficiency of AMT (40.0 mmol·L^–1^) ranges from 7.7% and 2.4% (for βCD-am_6_-βCD and βCD-am_12_-βCD, respectively) to approximately 60% when the amount of nanosponge increases from 1 to 50 mg. These results demonstrate that the two porous structures under study are effective in encapsulating AMT when the solutions are contacted with the CDNSs at a solid–liquid ratio (*R*_S/L_) of 50.

The loading capacity (LC), on the other hand, decreases by increasing the *R*_S‑L_ (see Table S5), which can be attributed to the lower amount of drug available in solution per gram of material, reducing the occupation of the encapsulation sites in the CDNSs.^36^ Nevertheless, even at *R*_S‑L_ = 50, both βCD-am_6_-βCD and βCD-am_12_-βCD can incorporate up to 146.1 mg (LC = 12.8%) and 149.2 mg (LC = 13.0%) of the drug AMT into their matrices, respectively. The EE and LC results highlight the strong affinity between AMT and the CDNSs, reflecting the effectiveness of these materials in retaining the drug within their structures.^50^

The encapsulation of AMT into the porous structures of the CDNSs was also confirmed by qualitative comparison of the SEM images for βCD-am_6_-βCD and βCD-am_12_-βCD before (Figure 6a,6b) and after (Figure 6c,6d) the adsorption process. Morphological changes in these materials are noticeable after contact with the aqueous AMT solution, as the micrographs prior to adsorption show a porous morphology, whereas after adsorption a marked reduction in the previously present pores is observed. This visible decrease in pore size corroborates the high adsorption efficiency determined in the adsorption studies discussed above.

**Figure 6:**
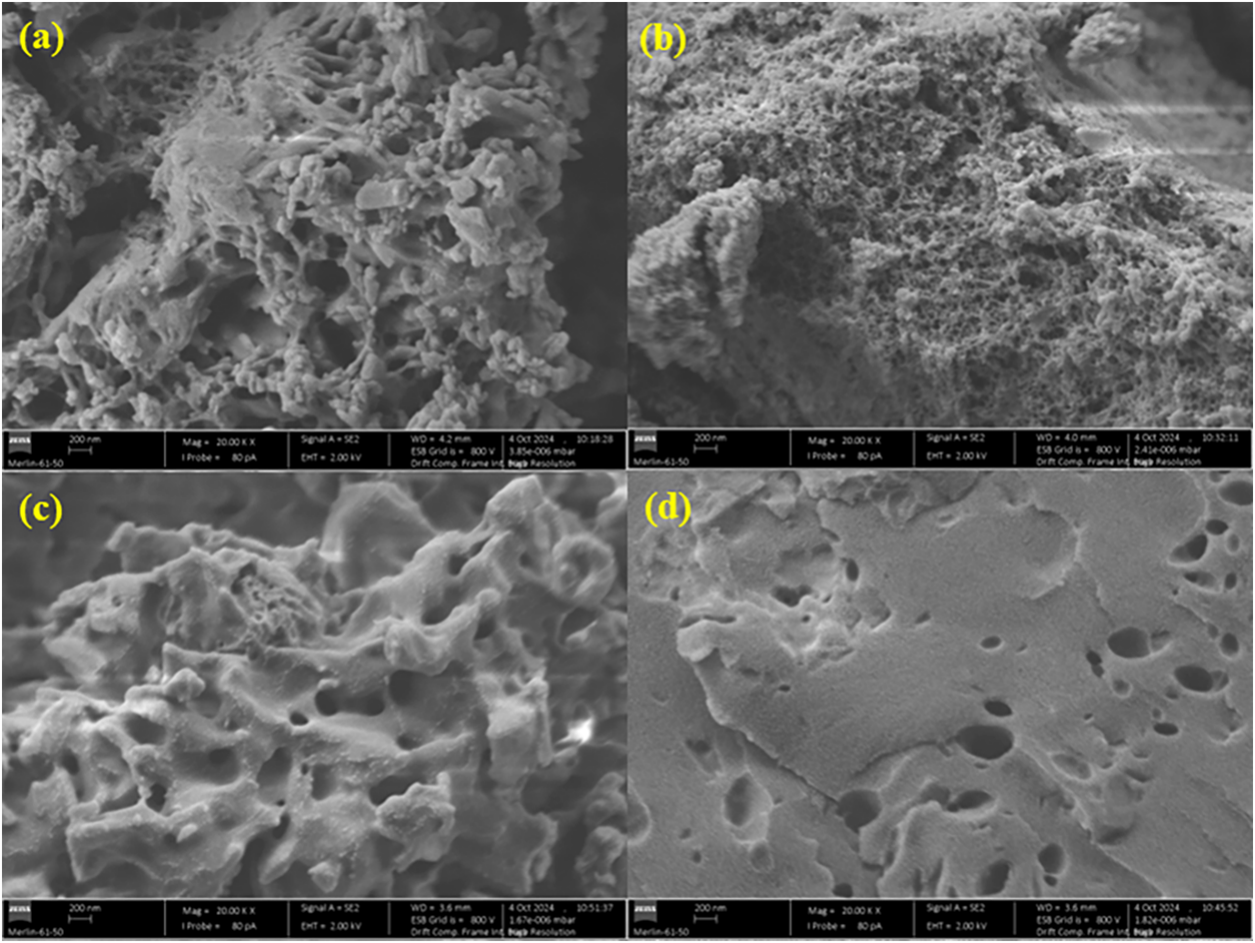
SEM micrographs obtained at ×20,000 magnification for βCD-am_6_-βCD (a) before and (c) after the adsorption process, and for βCD-am_12_-βCD (b) before and (d) after the adsorption process.

Once the encapsulation of AMT into the porous structures of the CDNSs was confirmed, the release of the drug was evaluated—a fundamental requirement for application in controlled drug delivery systems. A preliminary desorption assay was conducted by using 50.0 mg of the CDNSs previously loaded in the most efficient adsorption experiment. It can be observed (Figure 7b) that for both nanosponges, the release of AMT, in terms of removal efficiency (RE), is higher than 90%.

**Figure 7:**
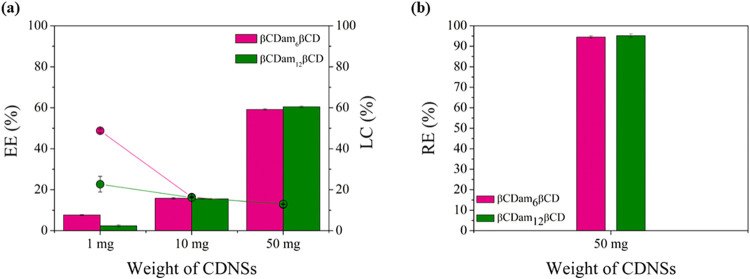
Graphs showing (a) encapsulation efficiency (bar) and loading capacity (line) of the drug AMT as a function of the weight of the materials and (b) release efficiency of the drug AMT, using 50.0 mg of each material.

Although the positive results for the use of β-CD-containing nanospoges in the sorption and desorption of AMT, no monoliths can be synthesized. Based on that, the next step has been focused on the development of a release device, using PVA hydrogels as the structural matrix.

### 3.3 Characterization of PVA Hydrogels

The structural characterization of the pure PVA hydrogels, obtained at two different concentrations, 14% and 20% (w/v), was carried out using FTIR-ATR spectroscopy, yielding results consistent with those previously reported in the literature, confirming the presence of the characteristic bands of the polymer.^51^ The IR spectra are shown in Figure S3, while the corresponding assignments are presented in Table S6. In addition, the surface morphology of both PVA hydrogels was evaluated, see Figure S4a,b, respectively. The 14% PVA hydrogel exhibits a porous structure characterized by larger and less dense pores compared with the 20% PVA hydrogel. This is due to the lower PVA concentration, which results in a less dense polymeric network that allows the formation of larger pores, whereas higher concentrations increase the density of the polymeric network, thereby reducing pore size.^52^ This is in close agreement with the swelling degree obtained for the hydrogels. From the swelling kinetics plotted in Figure 8, after ca. 1500 min (25 h) hours the swelling equilibrium is reached and the obtained swelling degree is higher for less concentrated matrix; i.e., *Q*_S_ are equal to (60 ± 3) and (30 ± 4), for PVA 14 and 20%, respectively. From these swelling degree values, the polymer volume fraction can be computed and is equal to 0.014 and 0.028,^53^ respectively.

**Figure 8:**
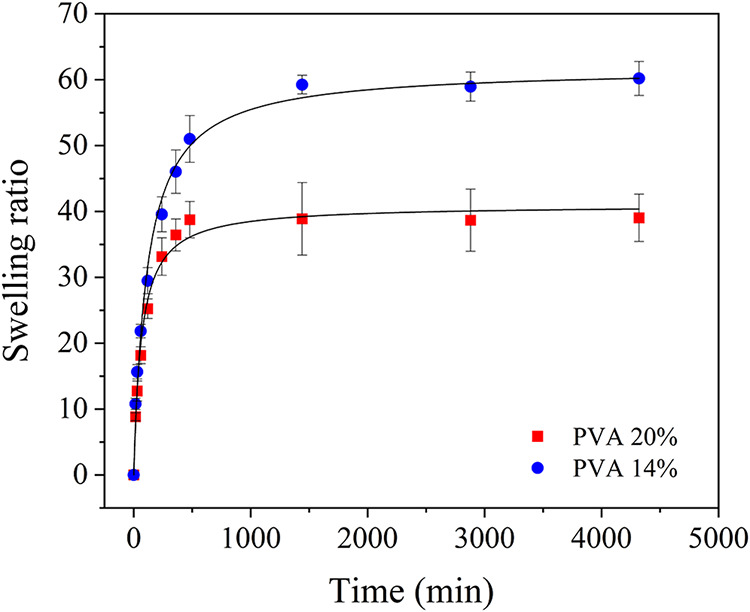
Swelling degree of the different hydrogels in water.

The density of the polymeric network formed by PVA exerts a direct influence on the swelling degree and, consequently, on the release profile, even if the water volume fraction is quite high.^52,54,55^ More compact and less porous structures limit the diffusion of the active agent and result in a slower and more controlled release, a desirable behavior in systems designed to maintain therapeutic drug concentrations over an extended period.^15^

To better understand the swelling mechanism of the hydrogels, pseudo-first-order and pseudo-second-order equations were used to model the experimental data. The equations can be described as follows

6 $$Q_{S,t}=Q_{S}\left(1-e^{-k_{1}t}\right)$$

7 $$Q_{S,t}=\frac{k_{2}Q_{S}^{2}t}{1+k_{2}Q_{S}t}$$

In eqs ([Disp-formula eq6]) and ([Disp-formula eq7]), *Q*_S,t_ is the swelling degree at time *t*, and *k*_1_ and *k*_2_ are the corresponding rate constants. From the analysis of fitting parameters summarized in Table 2, it comes out that the best fit corresponds to the application of Equation B, once *R* ^2^ and AIC values are always the highest and lowest ones, respectively. This behavior suggests that the swelling process is governed not only by water diffusion but also by the relaxation of polymer chains within the hydrogel matrix as a consequence of the possible formation of water clusters inside the gel phase.^56^ Evidence for the latter comes from the fact that *k*_2_ for the gel with a higher water volume fraction is lower than that found for the PVA 20%. This is explained when interactions water–water exceed polymer–water interactions and the obtained clusters act as obstacles to the diffusion process.^57^ Despite that, the swelling at short-range times (i.e., times corresponding to *Q*_S_/*Q*_S,t_ < 0.6) shows that the process is essentially pseudo-Fickian, justified by the high ability of both PVA matrices to sorb significant amounts of water. By applying the power law equation (eq 8),

8 $$Q_{S,t}/Q_{S}=kt^{n}$$

**Table 2:** Kinetic Parameters for Water Swelling 14 and 20% PVA Hydrogels

		materials	
equation	parameters	PVA 14%	PVA 20%
A	*q*_e_	57 ± 2	38.2 ± 0.8
	*k*_1_/min^–1^	(5.8 ± 0.8) × 10^–3^	(1.04 ± 0.09) × 10^–2^
	*R* ^2^	0.962	0.986
	AIC	16.7	8.44
B	*q*_e_	62 ± 1	40.96 ± 0.83
	*k*_2_	(1.4 ± 0.2) × 10^–4^	(3.8 ± 0.4) × 10^–4^
	*R* ^2^	0.992	0.990
	AIC	9.9	6.9
Power’s law	*k*	10 ± 2	11 ± 3
	*n*	0.22 ± 0.03	0.17 ± 0.04
	*R* ^2^	0.914	0.845
	AIC	21.1	20.1

where *k* is a proportional constant and *n* is related with the sorption mechanism, it can be computed that the best fitting of Equation (C) to experimental data leads to *n* values equal to 0.22 and 0.17, for PVA values below the 0.5—the limit value for a Fickian diffusion.^58^

### 3.5 Release Kinetics Study

Figure 9 shows the release profile of AMT from different polymeric matrices (see Figure S5 for all systems individually depicted). In addition, the TG and dTG data of PVA 20%/βCDam_6_βCD and PVA 20%/βCDam_12_βCD in the presence of the guest drug (AMT) are presented in Figure S6a,b, demonstrating the thermal stability below 100 °C. Studying the effect of PVA concentration on the AMT release (Figure S5a,b), the pseudo-first order (PFO) and pseudo-second order (PSO) kinetics equations were used to fit the experimental results. These equations were previously described as Equations (A) and (B) and, for the sake of simplicity, *Q*_s,t_ and *Q*_s_ are substituted by *q*_AMT,t_ and *q*_AMT,eq_, indicating the amount of AMT released at time *t* and at equilibrium, respectively. From the analysis of fitting parameters (Table S7), it can be seen that the AIC and *R* ^2^ values for the fitting of the PSO equation is better than those obtained for the PSO equation. However, the last one fails in the fitting of *q*_AMT,eq_ values, there is always a positive deviation, and without physical significance, from the experimental values (i.e., *q*_AMT,eq_ > 100%). Based on this, the Weibull equation (eq 9) was used as an alternative to successfully describe the overall profile of AMT release,^59,60^

9 $$q_{\mathrm{AMT},t}=q_{\mathrm{AMT},\mathrm{eq}}\left(1-e^{-k_{w}{\left(t-t_{0}\right)}^{d}}\right)$$

**Figure 9:**
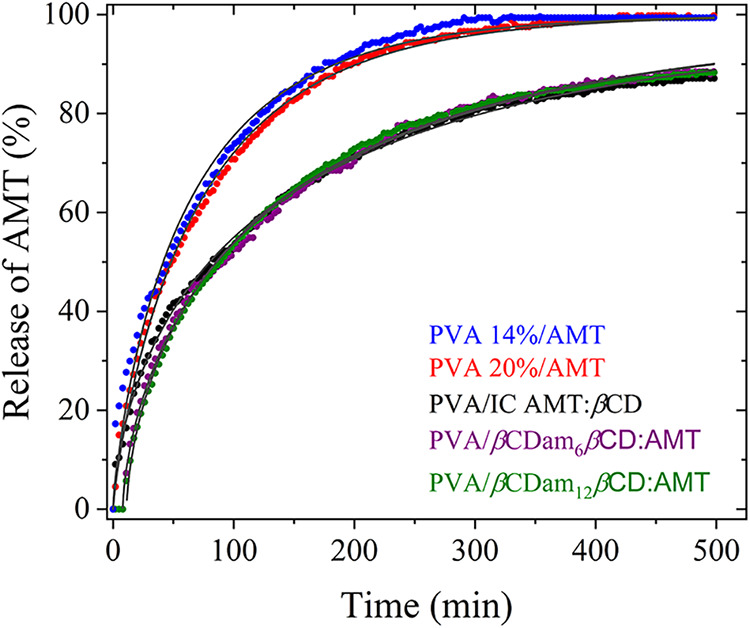
AMT release kinetics profiles from the following hydrogels: PVA 14%, PVA 20%, PVA 20%/IC, PVA 20%/βCDam_6_βCD, and PVA 20%/βCDam_12_βCD. For the sake of clarity, only the fitting of equation (C) to the experimental data is represented through solid lines.

where *k*_w_ defines the time scale of the process, *d* is a constant characterizing the shape of the release profile, and *t*_0_ represents the lag before the onset of the release process.^59^ The analysis of *d* values (Table 3) shows that the release mechanism is described by the diffusion in a normal Euclidean matrix with a further contribution^61^ which, in this case, might be the presence of PVA and/or the presence of water clusters. This result is again in agreement with the low polymer volume that exists in the PVA matrix. According to the Weibull model, *t*_50%_ and *t*_90%_ values were slightly higher for PVA20%/AMT (48 and 203 min, respectively) than for PVA14%/AMT (42 and 185 min, respectively), showing that increasing the polymer concentration prolongs the time required to reach 50 and 90% release, respectively.^59^ This trend is confirmed by the normalization of kw through the computation of MDT, where the following values are obtained: 272 min (PVA14%) and 342 min (PVA20%). The *t-*test (*p* < 0.05) confirmed the statistical difference between the two systems, which is consistent with the swelling results. These results evidenced that the increase of the density of the polymer matrix in the hydrogel with a higher PVA concentration acts as an effective barrier to drug diffusion, resulting in a slower and more controlled release. Thus, with the goal of developing a more controlled release system for AMT, 20% hydrogels were selected for the subsequent analyses.

**Table 3:** Fitting Kinetic Parameters Obtained by the Fitting Weibull Model Equation to the AMT Release from Different PVA-Based Hydrogels

		materials				
model	parameters	PVA 14%/AMT	PVA 20%/AMT	PVA/IC AMT:βCD	PVA/βCDam_6_βCD:AMT	PVA/βCDam_12_βCD:AMT
Weibull	*t*_0_/min	0	0	0	7.96 ± 0.08	11.2 ± 0.5
	*k*_w_/min^–d^	0.034 ± 0.002	0.027 ± 0.001	0.044 ± 0.001	0.041 ± 0.001	0.044 ± 0.001
	*d*	0.81 ± 0.01	0.836 ± 0.006	0.628 ± 0.003	0.651 ± 0.004	0.641 ± 0.005
	*R* ^2^	0.9896	0.9977	0.9981	0.9979	0.9981
	AIC	113.7	13.6	–19.8	4.6	0.7
	*t*_50%_/min	42 ± 3	48 ± 2	80 ± 2	86 ± 2	86 ± 3
	*t*_90%_/min	185 ± 10	203 ± 7	541 ± 14	500 ± 15	495 ± 22

By adding both the AMT-cyclodextrin and AMT-CD nanosponges, differences in the release profile can be observed, although in some cases, they may appear subtle. Thus, by adding the drug-containing complexes, it is possible to observe a significant decrease in the value of the *d* exponent, as well as a decrease in the percentage of AMT cumulatively released. Furthermore, the decrease of the value of *d* to values on the order of 0.63–0.65 suggests that the release of AMT is controlled by diffusion in heterogeneous systems, which is understandable considering the presence of host–guest complexes. These variations are accompanied by a significant increase in the values of *t*_50%_ and *t*_90%_ (when compared to cyclodextrin-free delivery systems).

Comparing the matrices with and without nanosponges, it can be seen that the presence of nanosponges leads to an attenuation of the burst release, with time lags increasing by increasing the alkyl chain length of the cross-linker’s nanosponge (*t*_0_ equal to 8 and 11 min, respectively); i.e., by increasing the intramolecular interactions, mainly hydrophobic,^25^ between the alkylchain in the nanosponge the more retained the drug is. In summary, the presence of CDNSs further attenuated the burst effect, since drug release depends not only on dissociation but also on diffusion through the cross-linked three-dimensional network. This supramolecular architecture, composed of multiple trapping sites, including both CD cavities and the pores of the polymeric matrix, contributes to the reduction of the burst effect and to a more controlled release.^62^

This indicates that AMT release is not governed solely by diffusion through the polymeric matrix, but also by specific interactions with βCD or CDNSs, mediated by inclusion and/or adsorption phenomena.^63,64^ Consequently, the release rate simultaneously depends on drug diffusion within the matrix and on the gradual dissociation of these interactions, resulting in a more controlled release profile (Figure 9). This behavior is consistent with the ability of CDNSs to retain the drug in their supramolecular network, as demonstrated by the adsorption experiments, thereby contributing to the modulation of release over time.

## 4 Conclusion

The hybrid polymer materials developed in this study demonstrate how supramolecular encapsulation can be integrated with polymer network transport to modulate drug delivery behavior. Physically cross-linked PVA hydrogels incorporating CDNSs were successfully prepared, yielding composite systems in which the polymer matrix acts as a diffusion-regulating scaffold, while CD’s cavities provide host–guest sites for drug inclusion. The presence of CDNSs modified the hydrogel microstructure and swelling behavior, indicating interactions between the dispersed supramolecular domains and the polymer network. This hierarchical architecture combines affinity-driven drug encapsulation with diffusion control, resulting in sustained release profiles compared with matrices lacking supramolecular hosts. Kinetic analysis indicates that drug release is governed by coupled mechanisms involving dissociation from CD’s cavities and diffusion through the hydrated polymer network. Overall, the incorporation of CDNSs into PVA hydrogels represents an effective strategy for designing hybrid materials with enhanced loading capacity and tunable release behavior, providing a versatile platform for advanced hydrogel-based drug delivery systems.

## Supplementary Material



## Notes

Inclusion complexes preparation methodology, kinetic equation models, FTIR spectra of βCD, I-βCD, am6, am12, and PVA hydrogels, table of infrared absorption bands, TG and dTG of βCD, I-βCD, PVA 20%/βCDam_6_βCD, and PVA 20%/βCDam_12_βCD, DLS measurements for βCD-am6-βCD and βCD-am12-βCD, data of EE%, LC, and LC%, SEM micrographs for PVA hydrogels, kinetic parameters for release of AMT, and AMT release kinetics profiles from the hydrogels (PDF)
