# Supplementary material for: Cyclodextrin Nanosponge–PVA Hybrid Networks as Supramolecular Reservoir Systems for Amitriptyline
Source: ACS Omega. 2026 Jul 16;11(29):43942–53. doi: 10.1021/acsomega.6c03745 (PMC13425283; doi:10.1021/acsomega.6c03745)
Supplement: Supplementary file 1 [file ao6c03745_si_001.pdf]

Supporting Information

Cyclodextrin Nanosponge–PVA Hybrid Networks as Supramolecular Reservoir Systems for Amitriptyline

Bianca B.M. Vieira[a], Pedro M.C Matias[b], Dina M.B. Murtinho[b], Eduardo Radovanovic[c], Frederico B. De Sousa[a], Artur J.M. Valente[b]**

[a] Laboratório de Sistemas Poliméricos e Supramoleculares (LSPS) – Instituto de Física e Química, Universidade Federal de Itajubá (UNIFEI), Itajubá, 37500-903, MG, Brazil.

[b] CQC-IMS, Department of Chemistry, Coimbra University, Coimbra, Portugal.

[c] Departamento de Química, Universidade Estadual de Maringá, Maringá, 87020-900, PR, Brazil.

Corresponding author:

Prof. Artur J. M. Valente
CQC-IMS, Department of Chemistry, Coimbra University, Coimbra, Portugal.
avalente@ci.uc.pt

Prof. Frederico B. De Sousa
Laboratório de Sistemas Poliméricos e Supramoleculares (LSPS) – Instituto de Física e Química, Universidade Federal de Itajubá (UNIFEI), Itajubá, 37500-903, MG, Brazil.
fredbsousa@gmail.com, fredbsousa@unifei.edu.br

Supplementary data S1

Inclusion complex preparation:

The inclusion compound in the solid state was prepared by freeze-drying method at 1:1 AMT:βCD molar ratio (Free Zone 4.5 Labconco). Aqueous solution of βCD, (88.1 μmol) and AMT (at 88.1 μmol) were prepared and then mixed. The mixture was stirred for 4 hours to ensure equilibrium between the host and guest molecules. Subsequently, the solution was frozen and subjected to freeze-drying for 48 hours at –51 °C.

Supplementary data – S2

Kinetic models (equations):

Pseudo-first order

$$q_t = q_e(1 - e^{-k_1 t})$$

Pseudo-second order

$$q_t = \frac{k_2 q_e^2 t}{1 + k_2 q_e t}$$

Peppas

$$q_t = kt^n$$

Supplementary data – S3

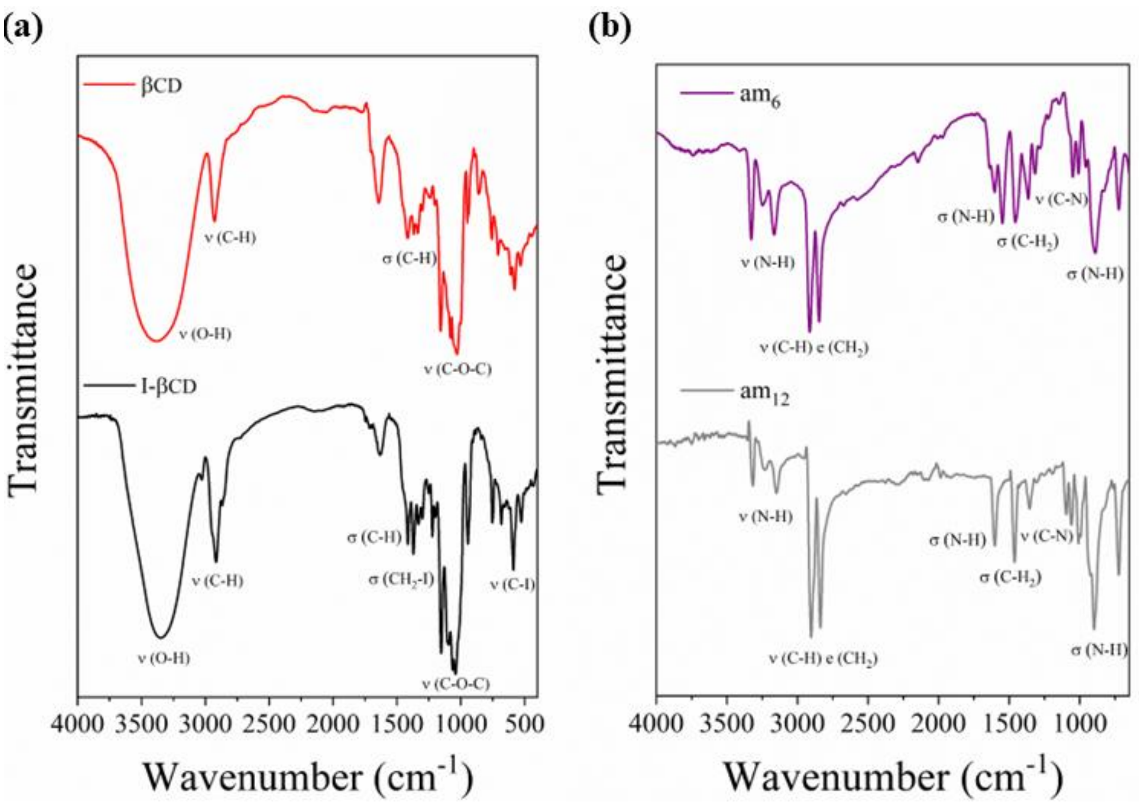

Figure S1: FTIR spectra of (a) βCD and I-βCD (KBr); and (b) am_6 and am_{12} (ATR).

Supplementary data – S4

Table S1: Infrared absorption band assignments for βCD and I-βCD.

Cyclodextrins	Regions (cm^{-1})	Assignments
βCD	3389	ν (O-H)
I-βCD	3348	
βCD	2925	ν (C-H)
I-βCD	2915	
βCD	1429 – 1214	δ (C-H)
I-βCD	1426 – 1217	
βCD	1034	ν (C-O-C)
I-βCD	1037	
I-βCD	1200	δ (CH_2-I)
I-βCD	586	ν (C-I)

Supplementary data – S5

Table S2: Infrared absorption band assignments for the amines am_6 and am_{12}.

Amine	Regions (cm^{-1})	Assignments
am_6	3327 – 3163	ν (N-H) primary amines
am_{12}	3324 – 3155	
am_6	29112 e 1847	ν (C-H) e ($C-H_2$) symmetric and asymmetric
am_{12}	2902 e 2847	
am_6	1602	δ (N-H) primary amines
am_{12}	1600	
am_6	1460	δ ($C-H_2$)
am_{12}	1463	
am_6	1354	ν (C-N)
am_{12}	1356	
am_6	880	Out-of-plane δ (N–H)
am_{12}	900	

Supplementary data – S6

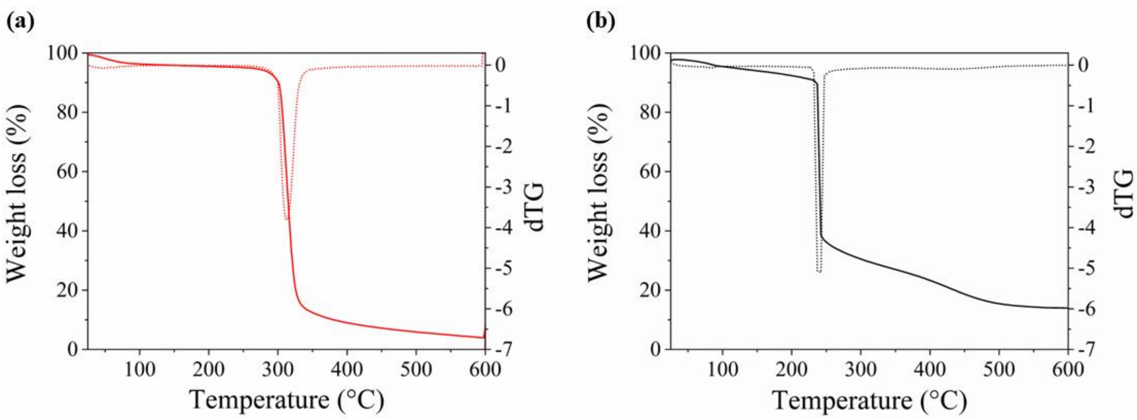

Figure S2: TG (solid line) and dTG (dash dot line) curves of (a) βCD and (b) I-βCD.

Table S3: Parameters obtained by DLS for βCD-am$_6$-βCD, at pH ranging from 2 to 12.

pH	Intensity (d.nm)	Intensity (%)	Number (d.nm)	Number (%)	PDI
**2**	434.80 ± 33.8	90.9 ± 0.9	409.8 ± 22.6	16.0 ± 2.7	0.6 ± 0.01
	123.6 ± 10.5	9.1 ± 0.9	114.5 ± 10.2	84.0 ± 2.7	
**3**	513.4 ± 22.2	91.4 ± 2.2	483.2 ± 21.4	12.4 ± 4.3	0.6 ± 0.04
	121.2 ± 19.0	8.6 ± 2.2	112.8 ± 17.1	87.6 ± 4.3	
**4**	425.1 ± 50.9	87.8 ± 7.8	387.4 ± 45.7	14.5 ± 4.4	0.5 ± 0.04
	124.6 ± 29.9	12.2 ± 7.8	114.6 ±26.2	85.5 ± 4.4	
**5**	499.8 ± 42.4	93.4 ± 0.6	482.3 ±41.5	8.4 ± 2.8	0.7 ± 0.03
	103.9 ±11.0	6.6 ± 0.6	100.0 ± 10.8	91.6 ± 2.8	
**6**	446.1 ± 31.8	100 ± 0.0	432.0 ± 30.1	100 ± 0.0	0.7 ± 0.05
**7**	399.5 ± 11.1	90.4 ± 5.9	378.4 ± 8.5	13.3 ± 1.1	0.6 ± 0.07
	122.2 ± 29.1	9.6 ± 5.9	114.0 ± 26.2	87.7 ± 1.1	
**8**	502.7 ± 17.5	91.0 ± 3.6	478.9 ± 14.9	10.9 ± 4.5	0.6 ± 0.02
	116.4 ± 23.7	9.0 ± 3.6	111.0 ± 21.7	89.1 ± 4.5	
**9**	1282.0 ± 6.4	100 ± 0.0	1270.5 ± 3.5	100 ± 0.0	0.2 ± 0.01
**10**	582.0 ± 20.8	100 ± 0.0	572.4 ± 21.1	100 ± 0.0	0.7 ± 0.03
**11**	434.5 ± 11.6	100 ± 0.0	412.8 ± 7.4	100 ± 0.0	0.8 ± 0.07
**12**	369.0 ± 31.1	100 ± 0.0	353.8 ± 26.0	100 ± 0.0	0.8 ± 0.06

Supplementary data – S8

Table S4: Parameters obtained by DLS for βCD-am$_{12}$-βCD, at pH ranging from 2 to 12.

pH	Intensity (d.nm)	Intensity (%)	Number (d.nm)	Number (%)	PDI
**2**	437.1 ± 97.9	100 ± 0.0	433.4 ± 96.3	100 ± 0.0	0.8 ± 0.04
**3**	1467.0 ± 355.7	100 ± 0.0	1336.5 ± 190.2	100 ± 0.0	0.9 ± 0.10
**4**	626.0 ± 19.9	100 ± 0.0	617.0 ± 25.5	100 ± 0.0	0.9 ± 0.01
**5**	665.3 ± 162.0	100 ± 0.0	654.0 ± 160.6	100 ± 0.0	0.9 ± 0.09
**6**	989.4 ± 403.9	93.7 ± 0.3	965.9 ± 376.4	3.9 ± 3.5	0.8 ± 0.06
	97.4 ± 26.3	6.3 ± 0.3	94.05 ± 26.5	96.1 ± 3.5	
**7**	1083 ± 381.0	84.2 ± 5.0	1028.5 ± 313.3	12.8 ± 2.1	0.9 ± 0.06
	181.1 ± 52.9	15.8 ± 5.0	171.8 ± 48.0	87.2 ± 2.1	
**8**	620.7 ± 21.5	90.1 ± 2.1	605.6 ± 20.1	4.7 ± 2.8	0.8 ± 0.04
	106.3 ± 19.9	9.9 ± 2.1	100.1 ± 18.1	95.3 ± 2.8	
**9**	857.4 ± 119.8	77.2 ± 5.4	843.0 ± 118.1	11.9 ± 1.1	0.9 ± 0.06
	185.6 ± 8.5	22.8 ± 5.4	174.5 ± 5.3	88,1 ± 1.1	
**10**	207.2 ± 40.3	100 ± 0.0	204.0 ± 37.7	100 ± 0.0	1.0 ± 0.00
**11**	663.0 ± 84.6	100 ± 0.0	651.7 ± 89.0	100 ± 0.0	1.0 ± 0.03
**12**	756.6 ± 199.1	90.3 ± 3.0	744.9 ± 203.5	7.2 ± 7.0	0.9 ± 0.05
	118.1 ± 43.1	9.7 ± 3.0	111.4 ± 42.0	92.8 ± 7.0	

Supplementary data – S9

Table S5: Encapsulation efficiency (EE%), loading capacity (LC), and loading percentage (LC%) of βCD-am$_6$-βCD and βCD-am$_{12}$-βCD materials at different solid-to-liquid ratios (R_{S-L}).

NECD	R_{S-L}	EE (%)	LC (mg. g^{-1})	LC%
βCD-am$_6$-βCD	1	2.38 ± 0.52	294.87 ± 64.16	22.68 ± 3.83
	10	15.55 ± 0.26	192.80 ± 3.21	16.60 ± 0.23
	50	60.47 ± 0.39	149.93 ± 0.96	13.04 ± 0.07
βCD-am$_{12}$-βCD	1	7.68 ± 0.26	952.66 ± 32.10	48.78 ± 0.84
	10	15.93 ± 0.39	196.20 ± 4.81	16.40 ± 0.34
	50	59.19 ± 0.39	146.76 ± 0.96	12.80 ± 0.07

Supplementary data – S10

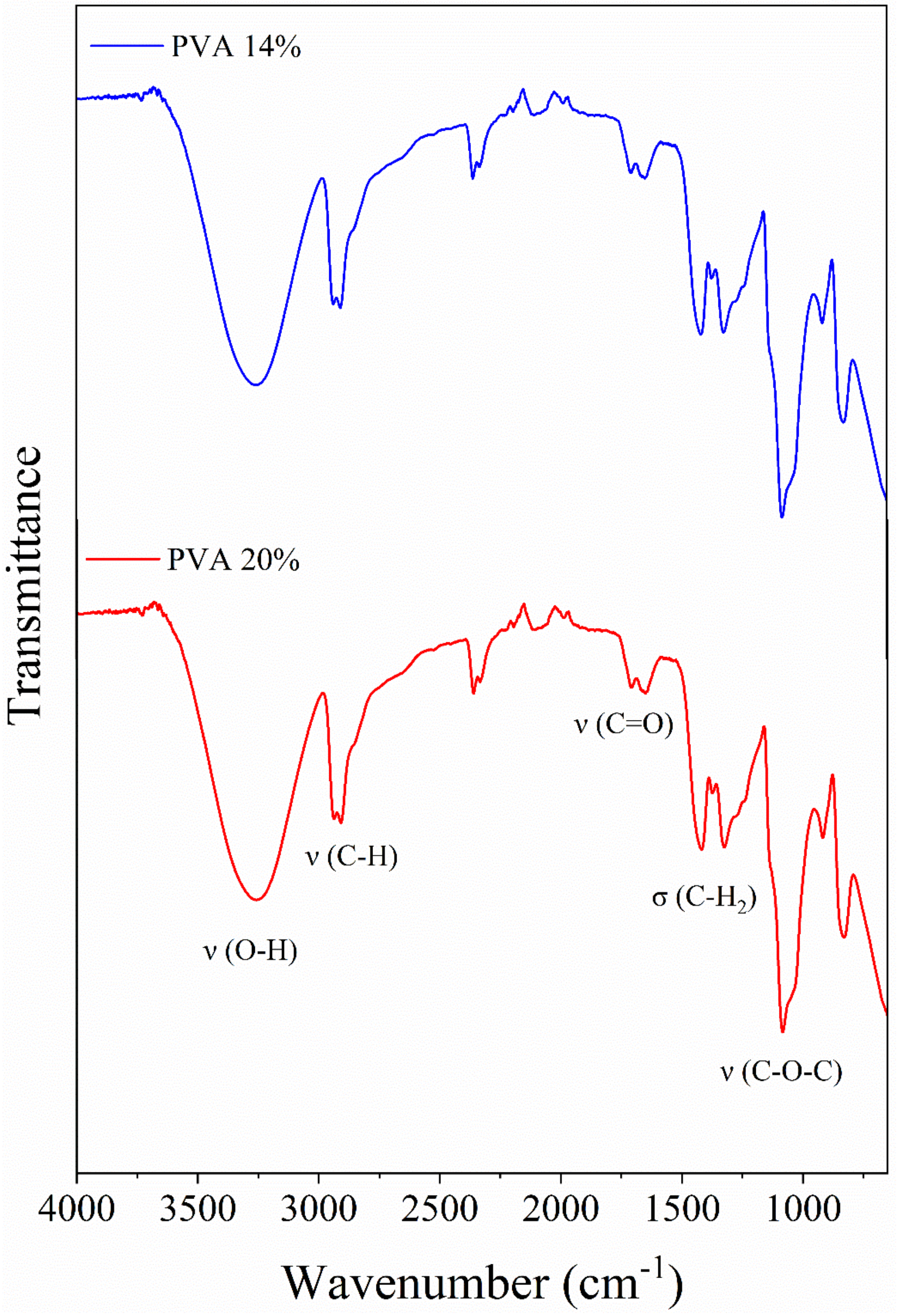

Figure S3: FTIR-ATR spectra of PVA hydrogels.

Supplementary data – S11

Table S6: Infrared absorption band assignments for the PVA.

**Regions (cm^{-1})**	**Assignments**
3260	ν (O-H)
2938 e 2910	ν (C-H)
1719	ν (C=O)
1418	δ (C-H_2)
1075	ν (C-O-C)

Supplementary data – S12

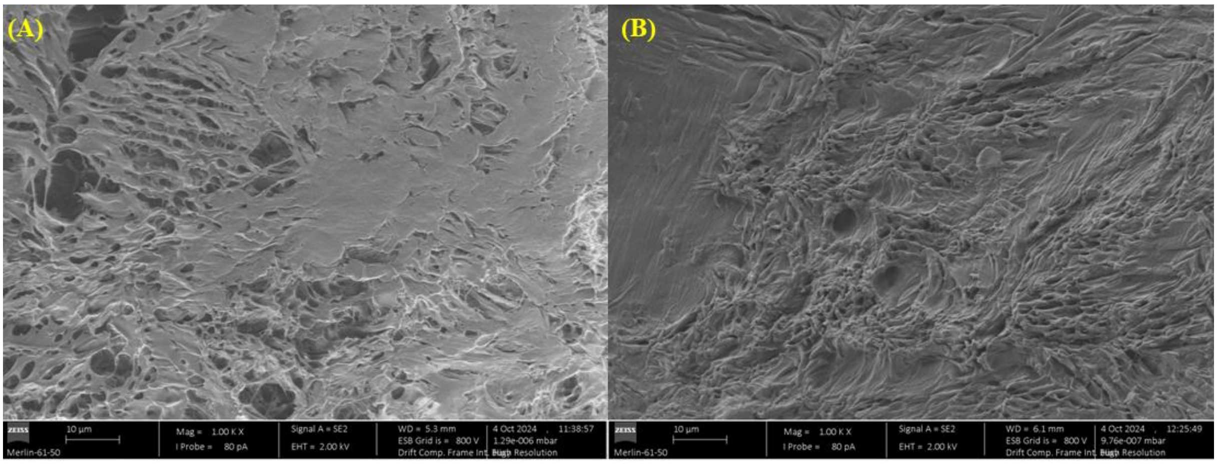

Figure S4: SEM micrographs obtained at 5,000× magnification for PVA hydrogels:

(a) 14%, and (b) 20%.

Supplementary data – S13

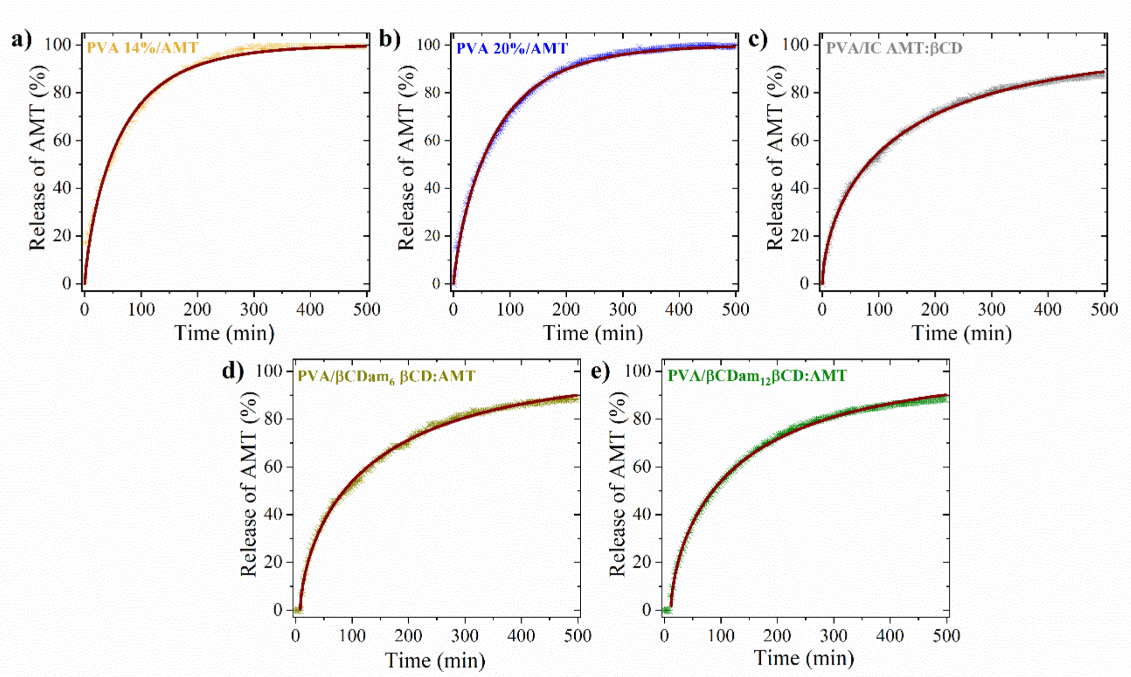

Figure S5: AMT release kinetics profiles from the following hydrogels: a) PVA 14%, b) PVA 20%, c) PVA 20%/IC, d) PVA 20%/βCDam6βCD and e) PVA 20%/βCDam12βCD. For the sake of clarity only the fitting of Equation (C) to the experimental data are represented through solid lines.

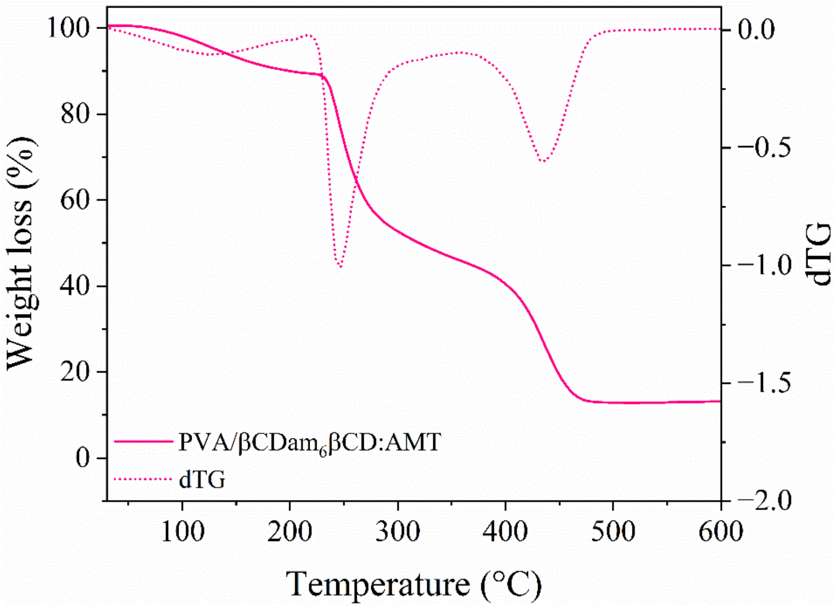

(a)

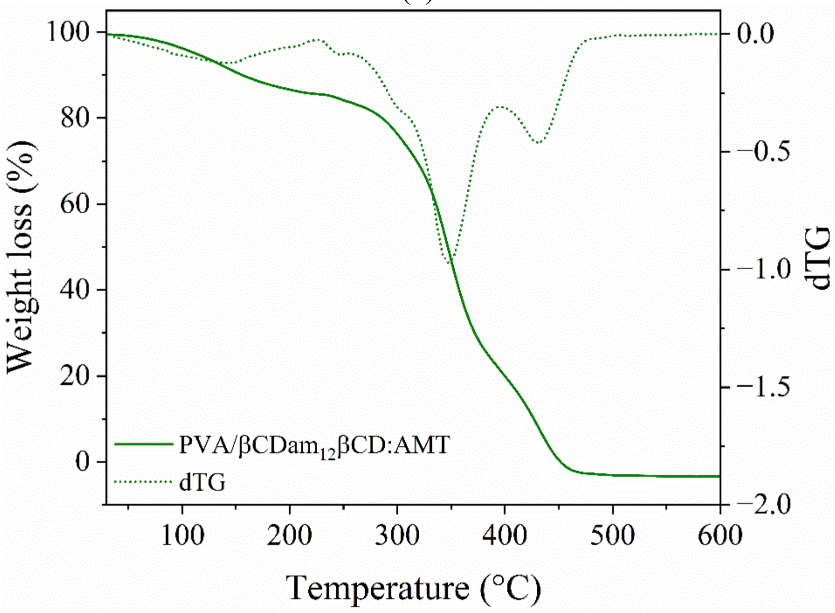

(b)

Figure S6: Thermograms (solid line) and corresponding derivatives (dTG, dash dot line) for (a) PVA 20%/βCDam6βCD and (b) PVA 20%/βCDam12βCD.

Table S7: Kinetic parameters for release of AMT.

Model	Parameters	Materials				
		**PVA 14%/AMT**	**PVA/AMT**	**PVA/IC AMT:βCD**	**PVA/βCDam$_6$βCD:AMT**	**PVA/βCDam$_{12}$βCD:AMT**
**Pseudo-first order**	q_e	99.22 ± 0.18	99.35 ± 0.14	88.44 ± 0.26	90.38 ± 0.20	90.45 ± 0.16
	k_1	0.015 ± 1.732 × 10^{-4}	0.013 ± 1.099 × 10^{-4}	0.009 ± 1.632 × 10^{-4}	0.008 ± 8.728 × 10^{-5}	0.008 ± 7.161 × 10^{-5}
	R^2	0.979	0.990	0.941	0.987	0.992
	AIC	229.23	155.55	278.42	202.28	151.27
**Pseudo-second order**	q_e	107.67 ± 0.34	109.09 ± 0.28	100.38 ± 0.22	104.16± 0.22	104.39 ± 0.20
	k_1	2.2 × 10^{-4} ± 5.6 × 10^{-6}	1.8 × 10^{-4} ± 3.5 × 10^{-6}	1.3 ×10^{-4} ± 1.6 ×10^{-6}	1.0 ×10^{-4} ± 1.3 ×10^{-6}	1.0 ×10^{-4} ± 1.1 ×10^{-6}
	R^2	0.971	0.985	0.994	0.995	0.997
	AIC	269.30	209.64	91,85	77.24	48.14
**Peppas**	K	32.07 ± 1.27	27.92 ± 1.16	17.06 ± 0.55	14.79 ± 0.61	14.70 ± 0.65
	N	0.1804 ± 0.0065	0.2022 ± 0.0068	0.2591 ± 0.0053	0.2840 ± 0.0067	0.2854 ± 0.0072
	R^2	0.812	0.841	0.936	0.918	0.910
	AIC	648.77	495.37	370.51	423.20	438.21
